# Cardiopulmonary Considerations for High School Student-Athletes During the COVID-19 Pandemic: NFHS-AMSSM Guidance Statement

**DOI:** 10.1177/1941738120941490

**Published:** 2020-07-09

**Authors:** Jonathan A. Drezner, William M. Heinz, Irfan M. Asif, Casey G. Batten, Karl B. Fields, Neha P. Raukar, Verle D. Valentine, Kevin D. Walter

**Affiliations:** † Department of Family Medicine, UW Medicine Center for Sports Cardiology, University of Washington, Seattle, Washington; ‡ National Federation of State High School Associations, Indianapolis, Indiana; § Department of Family and Community Medicine, University of Alabama at Birmingham, Birmingham, Alabama; ‖ Kerlan-Jobe Orthopaedic Institute, Los Angeles, California; ¶ Cone Health Sports Medicine, Greensboro, North Carolina; # Department of Emergency Medicine, Mayo Clinic, Rochester, Minnesota; ** Sanford Orthopedics & Sports Medicine, Sioux Falls, South Dakota; †† Departments of Orthopaedic Surgery & Pediatrics, Medical College of Wisconsin, Milwaukee, Wisconsin

**Keywords:** cardiac, coronavirus, COVID-19, prevention, screening, sports

SARS-CoV-2, the novel coronavirus that causes COVID-19 illness, presents unique health issues that should be considered in student-athletes prior to a return to sports and exercise. While the vast majority of young persons afflicted with the coronavirus have mild symptoms or remain asymptomatic, the infection can cause direct injury or inflammation to the heart and lungs, especially in patients ill enough to require hospitalization. Cardiopulmonary concerns from COVID-19 arise from data in severely ill adult patients, where approximately 1 in 5 hospitalized patients suffers from cardiac or thromboembolic (clotting) complications.^9,13^ However, evidence on the prevalence and risks of these complications in adolescents and in individuals who have had a milder form of the illness remains limited.^7^

An expert task force was formed from the National Federation of State High School Associations (NFHS) and the American Medical Society for Sports Medicine (AMSSM) to provide guidance for the medical assessment of student-athletes with prior COVID-19 illness before sports participation. The task force recommends that schools consider a supplemental questionnaire addressing medical issues specific to COVID-19 (Figure 1). Any positive response from the survey should trigger an evaluation by a medical provider prior to sports participation.

**Figure 1.**
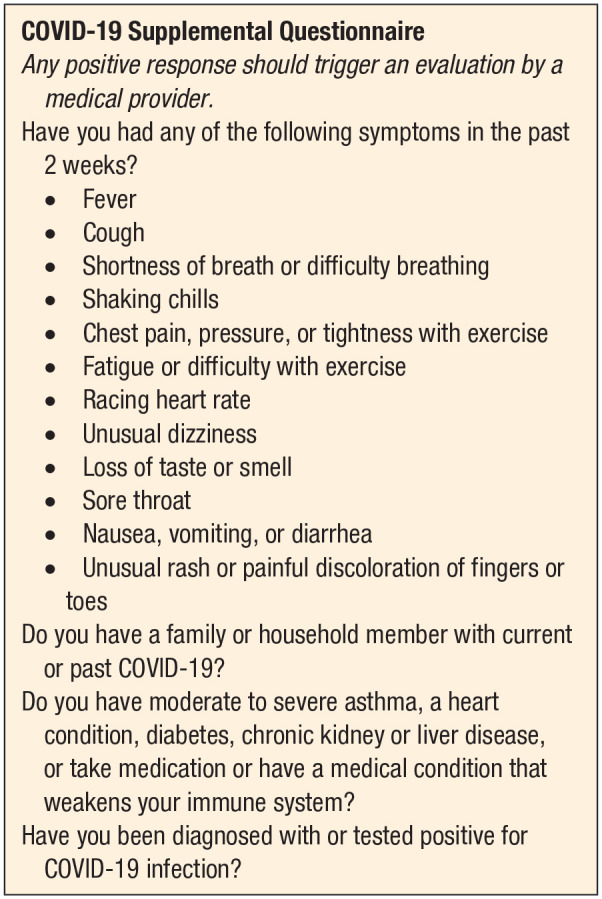
COVID-19 supplemental questionnaire.

## Prior COVID-19 Illness

**Figure 2.**
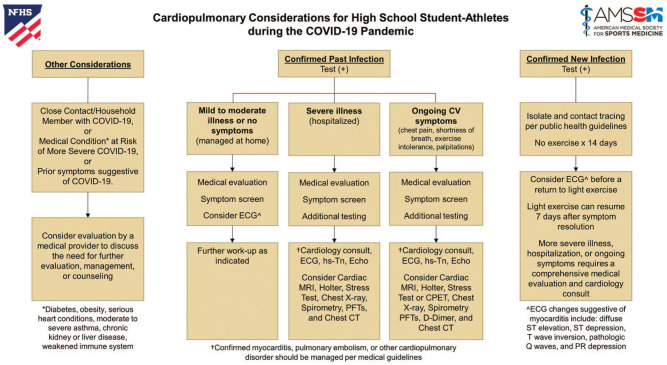
Cardiopulmonary considerations for high school student-athletes during the COVID-19 pandemic. CPET, cardiopulmonary exercise test; CT, computed tomography; CV, cardiovascular; ECG, electrocardiogram; Echo, echocardiogram; hs-Tn, high-sensitivity troponin; MRI, magnetic resonance imaging; PFT, pulmonary function test.

- *Confirmed diagnosis:* Every student-athlete with a prior confirmed diagnosis (positive test) for COVID-19 should undergo an evaluation by one’s medical provider prior to sports participation (Figure 2). Ongoing symptoms related to COVID-19 should be explored, including the presence of chest pain or shortness of breath with exertion, palpitations (heart racing), excessive fatigue, or decreased exercise tolerance. Written medical clearance is recommended prior to participation in sports.
- *Mild to moderate illness:* Student-athletes who had mild to moderate symptoms from COVID-19 that were managed at home should be seen by their medical provider for a detailed history of persisting symptoms or changes in their health status that may necessitate further testing or evaluation by a specialist.^1^ An electrocardiogram (ECG) may be considered prior to sports participation dependent on clinical suspicion for myocarditis (heart infection) and cardiology resources and expertise for ECG interpretation in young athletes.^1,2,12^
- *Severe (hospitalized) illness:* Student-athletes who were hospitalized with severe illness from COVID-19, including multisystem inflammatory syndrome in children, have a higher risk for heart or lung complications such as arrhythmias, myocarditis, heart failure, sudden cardiac arrest (SCA), and pulmonary embolism (blood clots to the lungs).^3^ A comprehensive cardiac evaluation is recommended in consultation with a cardiology specialist, which may include any or all of the following (as clinically indicated): ECG, cardiac biomarkers such as high-sensitivity troponin, echocardiogram, cardiac magnetic resonance imaging, Holter monitor, or stress test.^1,2,12^ Follow-up pulmonary evaluation and testing may also be indicated, including chest radiograph, spirometry, functional testing to assess bronchospasm or oxygen desaturation with exercise, chest computed tomography scan, or additional pulmonology consultation.
- *Ongoing symptoms:* Student-athletes with ongoing symptoms from diagnosed COVID-19 illness require a comprehensive evaluation to exclude heart and lung disorders that carry a risk of arrhythmia, respiratory compromise, SCA, or sudden death. Specifically, myocarditis may present with ongoing symptoms of chest pain, palpitations, shortness of breath, or exercise intolerance.^5,6^ Ongoing symptoms from COVID-19 may also indicate pulmonary issues such as acquired or worsening asthma, pneumonia, or pulmonary embolism that may cause chest pain, shortness of breath, tachycardia (fast heart rate), or a low oxygen saturation. The diagnosis of myocarditis, pulmonary embolism, or any other cardiopulmonary disorder should be managed per current medical guidelines. The student-athlete should not return to sports and/or exercise until medically cleared by a physician.^5,10^
- *Other considerations:* Evaluation by a medical provider should also be considered for the following circumstances:
  - Any “close contact” (ie, family or household member) with confirmed COVID-19 infection
  - Student-athletes with underlying medical conditions that place them at higher risk of severe COVID-19 illness, such as uncontrolled or moderate to severe asthma, a serious heart condition, obesity, diabetes, chronic kidney or liver disease, or a weakened immune system^8^
  - Student-athletes with prior symptoms suggestive of (but not confirmed) COVID-19, especially if symptoms were severe or required hospitalization

## New COVID-19 Infections

- Schools should consider a daily tracking tool to confirm student-athletes are appropriately self-monitoring and have not developed symptoms of COVID-19. In addition, it is suggested that schools establish a COVID-19 response team to help develop and implement policies and procedures for a safe return to sport in their school and to assist in COVID-19 symptom screening, reporting, and contact tracing.
- Student-athletes should not attend school, sports practices, or competitions if feeling ill and should be referred to their medical provider for possible COVID-19 testing if they present with any of the following symptoms: fever, new cough, difficulty breathing, shaking chills, chest pain, gastrointestinal symptoms (nausea, vomiting, or diarrhea), loss of taste or smell, sore throat, or an unusual rash or painful discoloration of fingers or toes.
- Student-athletes who test positive for COVID-19 with or without symptoms should be isolated per public health guidelines. No exercise is recommended for at least 14 days from diagnosis and 7 days after all symptoms have resolved.
- After symptom resolution and prior to sports participation, student-athletes should be evaluated by a medical provider to assess for residual symptoms and the need for additional testing. Written medical clearance prior to sports participation is recommended.

## Emergency Action Plan

Every school is reminded to have a well-rehearsed emergency action plan (EAP) for every sport at every venue to facilitate a coordinated and efficient response to SCA.^4,11,14^

- Every school should maintain an on-site automated external defibrillator (AED) program that allows retrieval and use of an AED within 3 minutes of collapse at school athletic venues and buildings.
- Potential first responders to SCA, including coaches, are encouraged to be trained in cardiopulmonary resuscitation (CPR), the recognition of SCA, and use of an AED.
- Each school should conduct and document an annual EAP practice drill for SCA among anticipated first responders (ie, athletic trainers, school nurses, coaches, and administrators).
- AED devices should be maintained according to manufacturer guidelines, including monthly readiness checks and scheduled battery or lead replacement.
